# Characteristics of Adults with Anxiety or Depression Treated at an Internet Clinic: Comparison with a National Survey and an Outpatient Clinic

**DOI:** 10.1371/journal.pone.0010885

**Published:** 2010-05-28

**Authors:** Nickolai Titov, Gavin Andrews, Alice Kemp, Emma Robinson

**Affiliations:** 1 School of Psychiatry, University of New South Wales, Sydney, Australia; 2 St Vincent's Hospital, Sydney, Australia; RIKEN Brain Science Institution, Japan

## Abstract

**Background:**

There is concern that people seeking treatment over the Internet for anxiety or depressive disorders may not resemble the general population or have less severe disorders than patients attending outpatient clinics or cases identified in community surveys. Thus the response to treatment in Internet based trials might not generalize.

**Methodology:**

We reviewed the characteristics of applicants to an Australian Internet-based treatment clinic for anxiety and depression, and compared this sample with people from a national epidemiological survey and a sample of patients at a specialist outpatient anxiety and depression clinic. Participants included 774 volunteers to an Internet clinic, 454 patients at a specialist anxiety disorders outpatient clinic, and 627 cases identified in a national epidemiological survey. Main measures included demographic characteristics, and severity of symptoms as measured by the Kessler 10-Item scale (K-10), the 12-item World Health Organisation Disability Assessment Schedule second edition (WHODAS-II), the Penn State Worry Questionnaire (PSWQ), the Body Sensations Questionnaire (BSQ), the Automatic Cognitions Questionnaire (ACQ), the Social Interaction Anxiety Scale (SIAS) and the Social Phobia Scale (SPS).

**Principal Findings:**

The severity of symptoms of participants attending the two clinics was similar, and both clinic samples were more severe than cases in the epidemiological survey. The Internet clinic and national samples were older and comprised more females than those attending the outpatient clinic. The Internet clinic sample were more likely to be married than the other samples. The Internet clinic and outpatient clinic samples had higher levels of educational qualifications than the national sample, but employment status was similar across groups.

**Conclusions:**

The Internet clinic sample have disorders as severe as those attending an outpatient clinic, but with demographic characteristics more consistent with the national sample. These data indicate that the benefits of Internet treatment could apply to the wider population.

## Introduction

Anxiety and depression are common mental disorders annually affecting approximately 20% of the adult population [1]–[3]. Over a 12 month period less than 40% of these people report seeking treatment from a health professional, with only a small percentage seeking treatment from a psychologist or psychiatrist.

One strategy for reducing barriers to treatment involves the development of Internet-based treatment programs for common mental disorders [4]. Such programs are based on cognitive behavioural therapy (CBT), are highly structured, and comprise online lessons, homework assignments, and regular communication with a therapist via email, telephone, or online forum [5]. The treatment efficacy or effectiveness of Internet-based CBT (ICBT) programs has been demonstrated for depression [6]–[8], panic disorder [9]–[12], generalized anxiety disorder (GAD) [13]–[14], and social phobia [15]–[23]. Typically, the benefits of ICBT with clinician-guidance is superior to self-guided programs, but on a public health scale self-guided programs are likely to be helpful, and both types are cost-effective relative to face-to-face treatment [24]. ICBT may reduce direct and indirect costs of treatment and increase access for people unable to find a local therapist, those unable to attend treatment during usual working hours, and those concerned about stigma [5]. The core question is “are people who seek Internet treatment different to people who seek face-to-face treatment or different to the average person in the population with the same disorder?”

The encouraging results from studies evaluating ICBT have triggered a rapid increase in “Internet clinics” (services providing ICBT or similar programs): For example, within the last 6 months at least 4 Internet clinics have begun operating in Australia. Given the medium, it is expected that Internet clinics will appeal mainly to younger people, however, little is known about the demographic characteristics of people who participate in online treatment. Such data are essential for determining the appeal and likely uptake of ICBT by different demographic groups, and will inform both decisions about the applicability of this form of treatment to a wider population, and how such services can be integrated with existing mental health services.

The present study reports the demographic characteristics and disorder severity of three groups of people: Participants at an Internet clinic operated at St Vincent's Hospital, Sydney; patients attending a specialist outpatient anxiety disorders clinic operated by the same hospital; and cases identified in a national epidemiological survey.

## Methods

This study was approved by the St Vincent's Hospital Human Research Ethics Committee. All participants provided written informed consent.

### Participants

The first group (the Internet clinic; IC group) comprised all participants in ICBT programs at the VirtualClinic (www.virtualclinic.org.au), treated between January 2008 and October 2009 (n = 774). The VirtualClinic is a joint venture between St Vincent's Hospital, Sydney, and the University of New South Wales. All participants had a DSM-IV [25] diagnosis of major depression, GAD, panic disorder, or social phobia confirmed via a structured diagnostic telephone interview using either the MINI 5.0.0 [26] or the CIDI 3.0 [27].

The second group comprised patients at a specialist Anxiety Disorders Clinic (the ADC group), operated by the Clinical Research Unit for Anxiety and Depression (CRUfAD), the same research and clinical team that manages the VirtualClinic at St Vincent's Hospital in Sydney, Australia. All people for whom data was available who had participated in group or individual treatment programs for GAD, panic disorder, or social phobia from January 2004 to March 2009, and who were over 18 years of age were included (n = 454). Diagnosis for ADC group participants was confirmed via a face-to-face interview with an experienced Consultant Psychiatrist or Clinical Psychologist.

The third group comprised respondents to the 2007 Australian National Mental Health Survey, a national epidemiological survey (the NS group), conducted by the Australian Bureau of Statistics between August and December 2007 to determine the prevalence of common mental disorders across Australia [28]. This survey included 8841 Australian residents aged 16–85 years (response rate of 60%) and derived data on lifetime mental disorders using the CIDI 3.0. We selected a subsample (unweighted) of respondents who met DSM-IV diagnostic criteria for lifetime depression, GAD, panic disorder, or social phobia and who reported having symptoms of that disorder in the last 12 months. In order to increase comparability with the clinic samples, if they had more than one disorder assessed in the survey, they were only included if they had chosen one of these four disorders as being the one that troubled them the most (n = 627). To maximise sample size, this subsample included respondents who reported seeking treatment (n = 324) as well as those who did not seek treatment (n = 303). Analyses confirmed that these two groups were similar on most of the variables examined in this study, except that those who sought treatment had significantly higher World Health Organisation Disability Assessment Schedule second edition (WHODAS-II) [29] scores (t = −2.5, p<0.05) and were more likely to have used the Internet for seeking mental health information or help (t = 44.8, p<0.001).

### Ethics

Data was obtained as part of the treatment of the two clinic groups and was de-identified before being accessed. Data on the survey participants was obtained from a de-identified unit record file supplied by the Australian Bureau of Statistics. Approval for the study was obtained from the Human Research Ethics Committee of St Vincent's Hospital. All participants provided written informed consent.

### Measurements

Demographic variables available for each group included age, gender, marital status, highest educational qualification, and employment status. Information about use of the Internet for mental health help or information was available for both the IC and NS groups. Kessler 10-item scores (K-10) [30], a measure of psychological distress, and the WHODAS-II scores, a general measure of disability, were available for all three groups. For people in the IC and ADC groups, the same disorder specific measures are reported when available, obtained at pre-treatment assessment. For people with a primary diagnosis of GAD the available measure was the Penn State Worry Questionnaire (PSWQ) [31], for those with panic disorder the measures included the Body Sensations Questionnaire (BSQ) [32] and the Agoraphobic Cognitions Questionnaire (ACQ) [32], and for those with social phobia the available measures were the Social Interaction Anxiety Scale (SIAS) and Social Phobia Scale (SPS) [33].

### Statistical Methods

Differences between samples in the categorical demographic variables were assessed using chi-square tests, while mean differences in age and the symptom severity scales were assessed using one-way ANOVAs. Post-hoc tests were conducted using chi-square tests for categorical variables and *t*-tests for continuous variables. Logistic regression analyses and ANCOVAs were used with the categorical and continuous variables, respectively, to investigate whether the results differed after controlling for age. Participants with missing data were omitted from specific analyses where the missing values occurred. Analyses were performed using the Statistical Package for Social Sciences (SPSS) version 17·0 for Windows [34], and the SAS-callable SUDAAN software package [35].

## Results

### Recruitment

The IC sample was recruited between January 2008 and October 2009; the ADC sample was recruited between January 2004 and May 2009; and the NS sample was recruited between August and December 2007.

### Demographic Characteristics

Demographic characteristics of people in the 3 samples are displayed in Table 1.

**Table 1:** Comparison of demographic characteristics of the Internet Clinic (IC), Anxiety Disorders Clinic (ADC), and the National Survey (NS) samples.

Category	Subcategory	*p*-Value	Test Statistic	IC (N)	IC *Mean (SD)/Percent*	ADC (N)	ADC *Mean (SD)/Percent*	NS (N)	NS *Mean (SD)/Percent*
***Mean Age (SD)***		<0.0005	F = 96.8	773	43.0 (12.7)*	454	32.8 (9.5)* ‡	627	42.0 (15.3)‡
***Age in Categories (%)***		<0.0005	χ2 = 238.3	773		454		627	
	18–24 years				8.2		21.6		16.1
	25–34 years				19.3		41.2		19.3
	35–44 years				27.9		25.8		24.1
	45–54 years				23.5		8.1		18.8
	55–64 years				17.6		3.1		13.1
	65+ years				3.5		0.2		8.6
***Gender (% male)***		<0.0005	χ2 = 26.4	773	33.8*	454	48.0* ‡	627	35.9‡
***Marital Status (%)***		<0.0005	χ2 = 191.0	772		447		627	
	Single/Never Married				30.4* †		60.9* ‡		40.5† ‡
	Married/Defacto				54.1		33.8		31.9
	Separated/Divorced/Widowed				15.4		5.4		27.6
***Highest Educational qualification (%)***		<0.0005	χ2 = 99.2	772		361		627	
	No qualification/High school				24.0†		26.0‡		45.3† ‡
	Vocational qualification/other certificate				17.2		15.8		19.3
	Diploma/Degree or above				58.8		58.2		35.4
***Employment status (%)***		0.311	χ2 = 2.3	772		447		627	
	Employed full-time or part-time				68.4		64.9		65.1
	Unemployed/Not working				31.6		35.1		34.9
***Ever used Internet for MH help/info (%)***		<0.0005	χ2 = 180.2	772	61.3†	-	-	627	25.4†

*Significant difference IC and ADC samples in follow-up t-tests;

† Significant difference between IC and NS samples in follow-up t-tests;

‡ Significant difference between ADC and NS samples in follow-up t-tests.

#### Age

The mean age of people in the IC (43.0 years) and NS (42.0 years) samples was significantly greater than those in the ADC sample (32.8 years) (*F* = 96.8, p<0.001). More than 40% of the IC and NS samples were over the age of 44, compared to 11% of the ADC sample.

#### Gender

There were more females in the IC (66%) and NS (64%) samples than in the ADC sample (52%) (*X* ^2^ = 26.4, p<0.001).

#### Marital Status

There were significant differences in marital status between samples (*X* ^2^ = 191.0, p<0.001): More people in the ADC sample were likely to be single compared to the NS sample who, in turn, were more likely to be single than those in the IC sample.

#### Highest Educational Qualification

There were significant differences in educational qualifications between samples: The IC and ADC samples had more people with higher qualifications than the NS sample (*X* ^2^ = 99.2, p<0.001).

#### Employment Status

No differences were observed in employment status (*X* ^2^ = 2.3, p = ns) across groups.

#### Use of the Internet for Mental Health Help/Information

The IC sample (61%) was more likely than the NS (25%) sample to report using the Internet for accessing information or help about mental health (*X* ^2^ = 180.2, p<0.001). This question was not asked of the ADC group.

### Disorder Scores

Disorder scores of people in the 3 samples are included in Table 2.

**Table 2:** Comparison of the symptom severity scores of the Internet Clinic (IC), Anxiety Disorders Clinic (ADC), and the National Survey (NS) samples.

Disorder	Scale	Test Statistic	*p*-Value	IC (N)	IC Mean (SD)	ADC (N)	ADC Mean (SD)	NS (N)	NS Mean (SD)
***General***					*n = 774*		*n = 454*		*n = 627*
	K-10	F = 330.9	<0.0005	709	26.1 (7.4) * †	422	33.2 (7.6) * ‡	627	21.1 (7.5) † ‡
	WHODAS-II	F = 164.0	<0.0005	677	14.7 (8.3) * †	418	12.1 (7.4) * ‡	626	7.0 (7.2) † ‡
***GAD***					*n = 230*		*n = 65*		
	PSWQ	F = 1.1	0.295	230	64.3 (10.0)	57	62.7 (10.5)	-	-
***Social Phobia***					*n = 271*		*n = 255*		
	SPS	F = 13.1	<0.0005	247	34.4 (15.8)	244	39.6 (15.5)	-	-
	SIAS	F = 0.5	0.471	247	54.3 (11.3)	244	55.1 (12.9)	-	-
***Panic Disorder***					*n = 85*		*n = 134*		
	BSQ	F = 1.6	0.208	64	52.7 (13.5)	128	49.9 (14.4)	-	-
	ACQ (nervous)	F = 2.3	0.129	64	32.8 (9.1)	128	35.2 (10.4)	-	-

*Significant difference between IC and ADC samples in follow-up t-tests;

† Significant difference between IC and National Survey samples in follow-up t-tests;

‡ Significant difference between ADC and NS samples in follow-up t-tests.

#### K-10

Significant differences in distress were found between samples (*F* = 330.9, p<0.001): Post-hoc tests revealed the ADC sample (33.2) had significantly higher K-10 scores than the IC sample (26.1) who, in turn, had significantly higher K-10 scores than the NS sample (21.1).

#### WHODAS-II

Significant differences in disability were found between samples (*F* = 164.0, p<0.001): Post-hoc tests revealed the IC sample (14.7) had significantly higher WHODAS-II scores than the ADC sample (12.1) who, in turn, had significantly higher WHODAS-II scores than the NS sample (7.0).

#### Generalized Anxiety Disorder

No difference was found in pre-treatment PSWQ scores between the IC and ADC samples (*F* = 1.1, p = ns). This measure was not administered to the NS group.

#### Social Phobia

The ADC sample had significantly greater SPS scores than the IC sample (*F* = 13.1, p<0.001). No difference was found in pre-treatment SIAS scores (*F* = 0.5, p = ns). This measure was not administered to the NS group.

#### Panic Disorder

No difference was found in pre-treatment BSQ and ACQ scores between the IC and ADC samples (*F* = 1.6–2.3, p = ns). This measure was not administered to the NS group.

#### Effect of Age

All analyses were repeated controlling for age, but no differences were found in the pattern of results described above.

## Discussion

Does the Internet group differ in terms of demographic characteristics? The Internet group were of similar age and gender distribution to the national sample, more likely to be married and more likely to have higher qualifications, but as likely to be employed. Compared to the clinic sample, the Internet group were older, less likely to be male, less likely to be married but equally well educated and as likely to be employed. We conclude that the Internet group are largely representative of the wider population of those with anxiety and depression.

Does the Internet group differ in terms of disorder severity? The national sample had the lowest distress and disability scores. The Internet group was less distressed but more disabled than the clinic group. People who apply for Internet treatment do not have mild variants of anxiety and depressive disorders. The Internet groups' panic, social phobia and generalized anxiety scores were comparable to the clinic group. We conclude that the Internet group have symptom severity scores comparable to those who seek face-to-face treatment.

The data from the National Survey reflect people with anxiety and depression in the Australian population and the measures used are valid. Similar measures were also used in the two clinic groups, and validated clinical measures were used to determine illness severity.

### Limitations

The number of participants was substantial but these findings require replication. The patients in the outpatient clinic reflect the catchment area from where they are drawn and samples from other clinics could have different demographic characteristics. The Internet clinic sample represent the people who applied for treatment during the first two years that the clinic operated. They may not prove to be representative of people who apply for Internet therapy.

### Conclusions

Applicants to an Internet clinic were similar in age, gender and employment status to those identified by a national epidemiological survey as meeting diagnostic criteria for anxiety or depressive disorders in the Australian community. The severity of distress, disability and disorder symptoms of the Internet sample was greater than that of the national sample but was similar to people attending a specialist outpatient treatment facility. These data have implications for policy makers and funding bodies. People seeking online treatment have substantial disorders and are not necessarily young and technologically sophisticated. They may represent a population who are mature, have a long history of illness experience, are motivated to seek and participate in treatment, but have had difficulty accessing traditional outpatient clinics.
